# Supplementary material for: Immune cellular networks underlying recovery from influenza virus infection in acute hospitalized patients
Source: Nat Commun. 2021 May 11;12:2691. doi: 10.1038/s41467-021-23018-x (PMC8113517; doi:10.1038/s41467-021-23018-x)
Supplement: Supplementary file 1 — Supplementary Information [file 41467_2021_23018_MOESM1_ESM.pdf]

SUPPLEMENTARY INFORMATION

Immune cellular networks underlying recovery from influenza virus infection in acute hospitalized patients

Thi H. O. Nguyen[1], Marios Koutsakos[1], Carolien E. van de Sandt[1,2], Jeremy Chase Crawford[3], Liyen Loh[1], Sneha Sant[1], Ludivine Grzelak[4], Emma K. Allen[3], Tim Brahm[3], E. Bridie Clemens[1], Maria Auladell[1], Luca Hensen[1], Zhongfang Wang[1], Simone Nüssing[1], Xiaoxiao Jia[1], Patrick Günther[1], Adam K. Wheatley[1], Stephen J. Kent[1,5,6], Malet Aban[7], Yi-Mo Deng[7], Karen L. Laurie[7,8], Aeron C. Hurt[7], Stephanie Gras[9-11], Jamie Rossjohn[9,10,12], Jane Crowe[13], Jianqing Xu[14], David Jackson[1], Lorena E. Brown[1], Nicole La Gruta[9], Weisan Chen[11], Peter C. Doherty[1], Stephen J. Turner[15], Tom C. Kotsimbos[16,17], Paul G. Thomas[3], Allen C. Cheng[18,19*#] and Katherine Kedzierska[1*#]

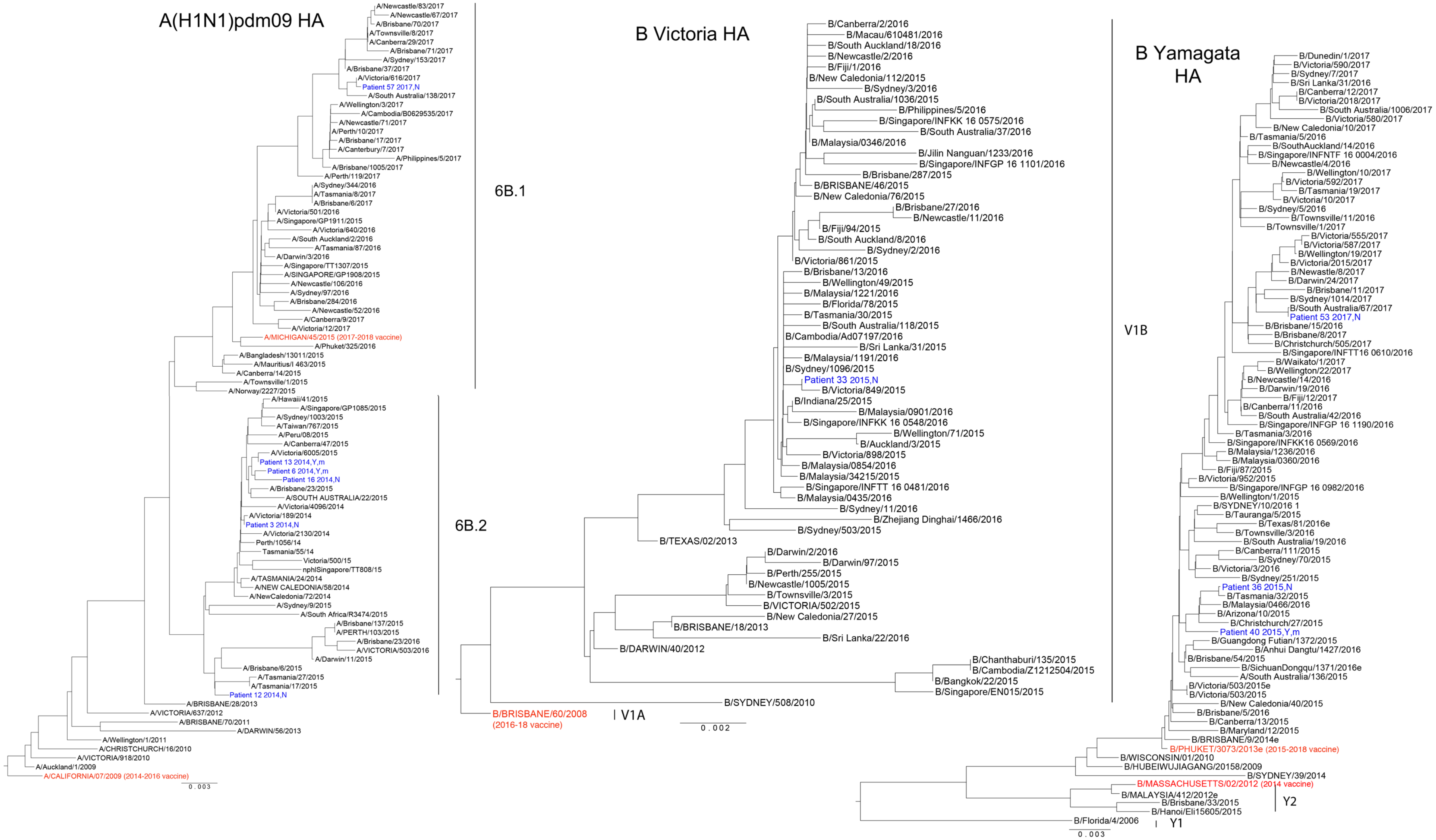

Supplementary Figure 1. HA phylogenetic trees for H1 and B strains. WHO reference strains are shown in black, influenza vaccine strains are in red, and sequences isolated from the nasal swab of patients are in blue. Patient number is followed by the year of recruitment, yes (Y) or no (N) for prior vaccination in the year of infection, and "m" for vaccine match. Scale bars represent the number of substitutions per site.

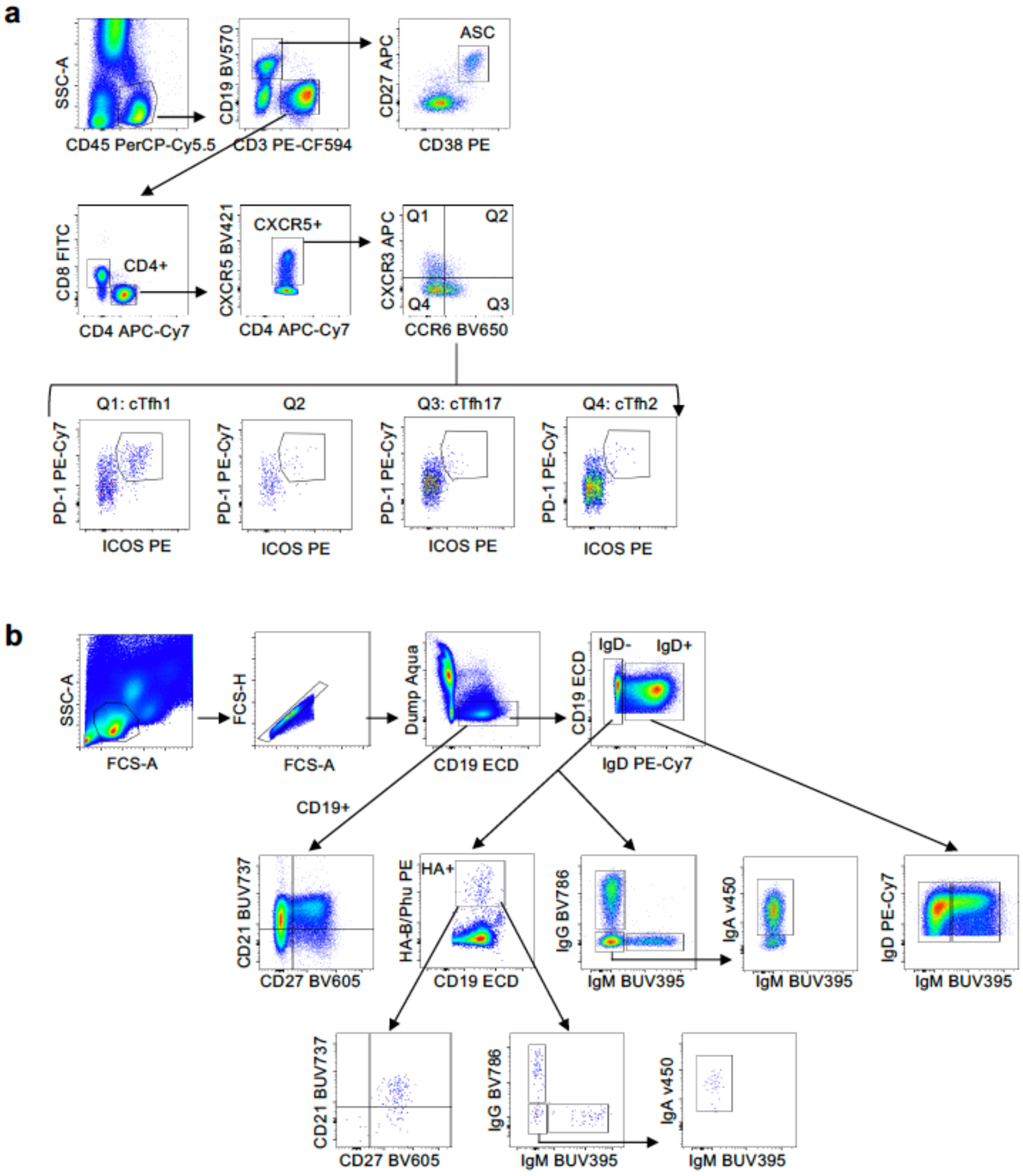

Supplementary Figure 2. Flow cytometry gating strategies for (a) cTfh, ASC (Fig. 3) and (b) HA-specific B cells (Fig. 4).

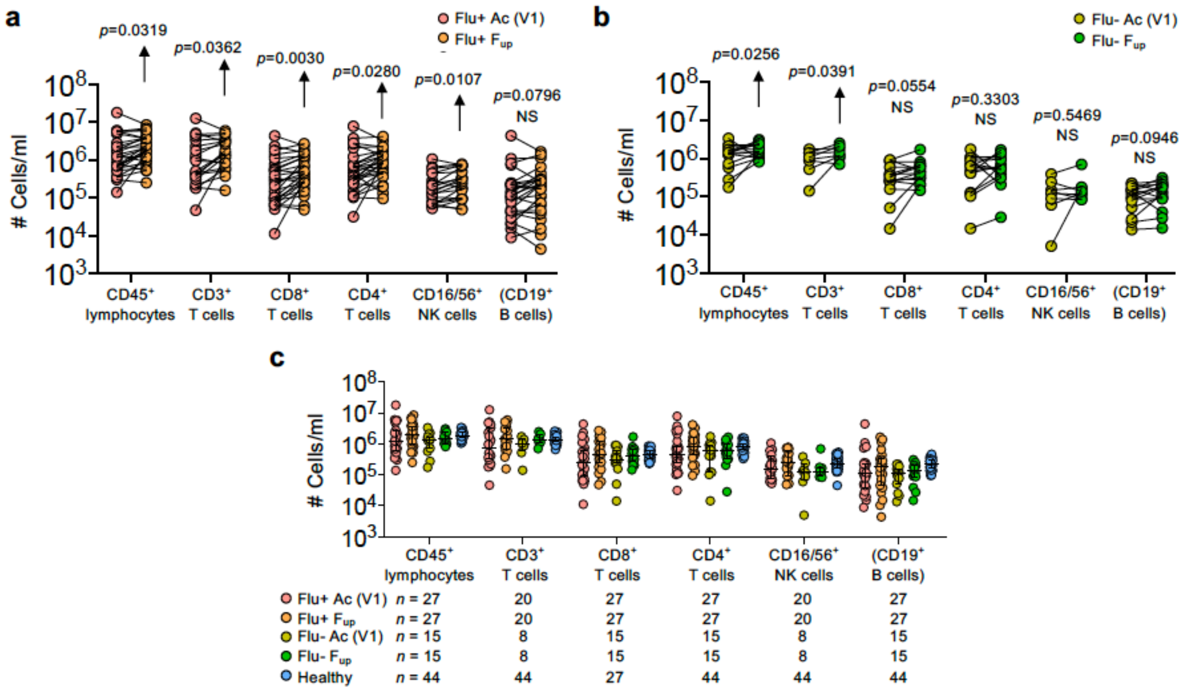

Supplementary Figure 3. Lymphopenia observed in patients during acute infection. a,b, Absolute numbers of cell subsets at acute (V1) and matching follow-up time-points in influenza+ (Flu+) and influenza- (Flu-) patients, where available. Statistical significance (0.0001>p<0.05) was determined using Wilcoxon test (two-sided) between acute and follow-up. **c**, Absolute numbers of cell subsets at acute (V1) and follow-up time-points in influenza+ patients in comparison to influenza- and healthy donors from the 2015-2016 pre-vaccinated cohort. Median, IQR and exact n numbers are shown for each group.

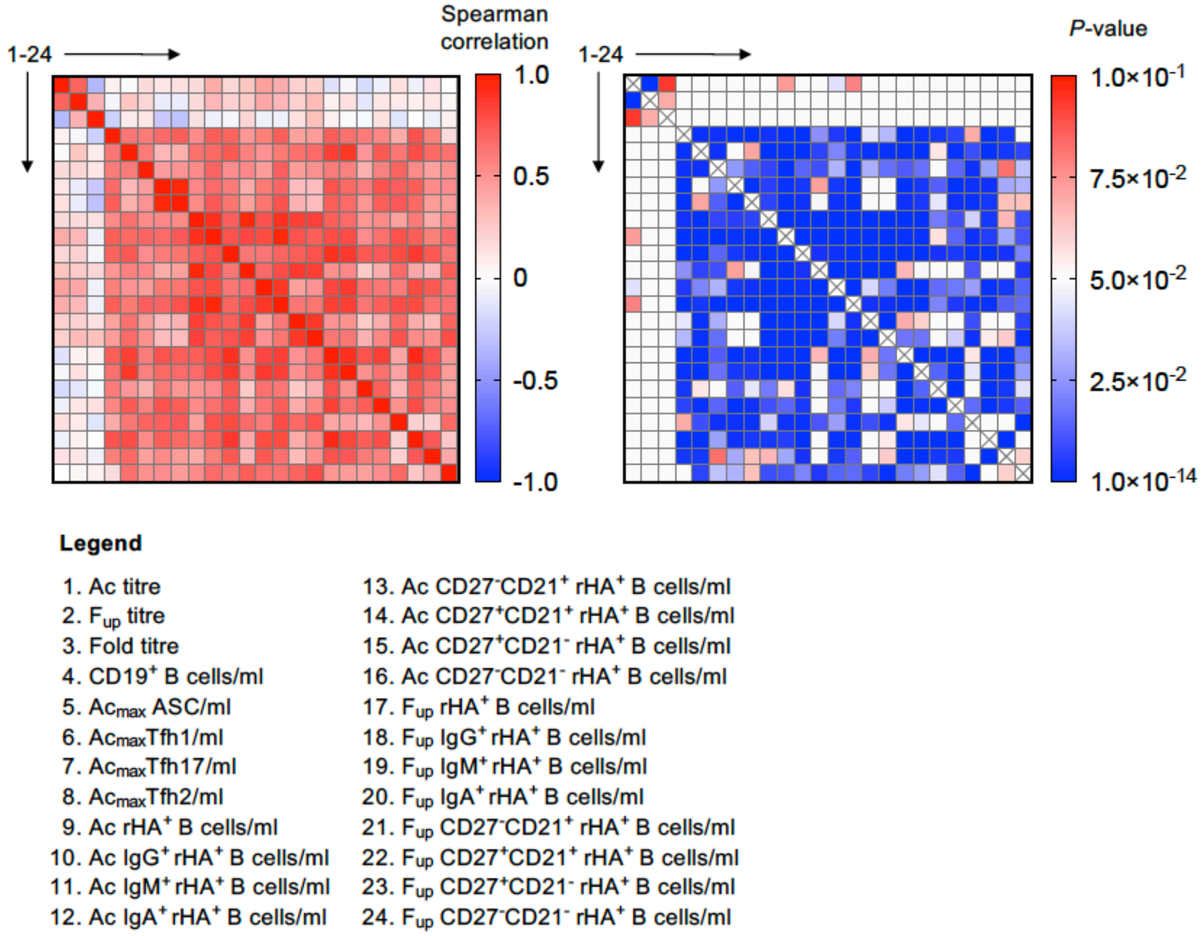

Supplementary Figure 4. Correlation matrix of antibodies, ASCs, cTfhs and HA-specific B cells. Spearman's correlation (two-sided) was calculated for each parameter and *p*-values ($1x10^{-14}>p<0.1$) for each correlation are plotted on the right. Only patients with acute HA-probe data sets were included in the analysis (n=29).

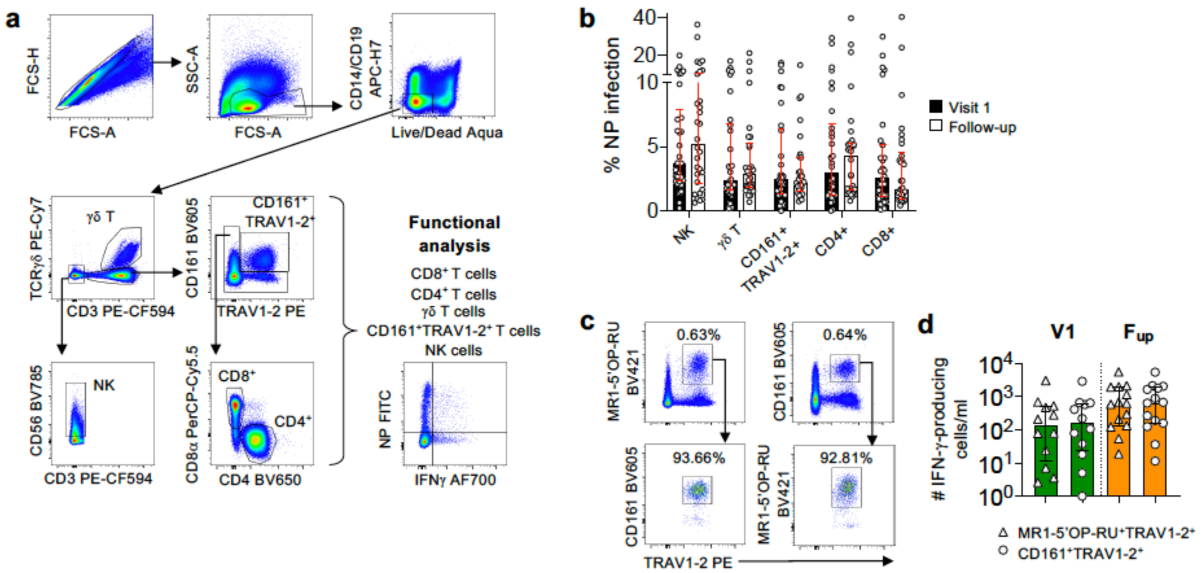

Supplementary Figure 5. Functional assessment of innate and adaptive immune cells. a-d, Data following influenza virus infection assay. **a**, FACS gating strategy measuring IFN-γ production from all cell subsets related to Fig. 5a-d. **b**, Frequency of intracellular NP staining per cell subset (*n*=30). **c**, Comparative FACS staining of MAIT cells, gated on live/CD19$^-$/CD14$^-$/CD3$^+$/TCRγδ$^-$ cells, using MR1-5'OP-RU-tetramer versus CD161 as surrogate markers for TRAV1-2$^+$ MAIT cells. **d**, Numbers of IFN-γ-producing MAIT cells using both surrogate markers (*n*=17). **b,d**, Median bars and IQR are shown.

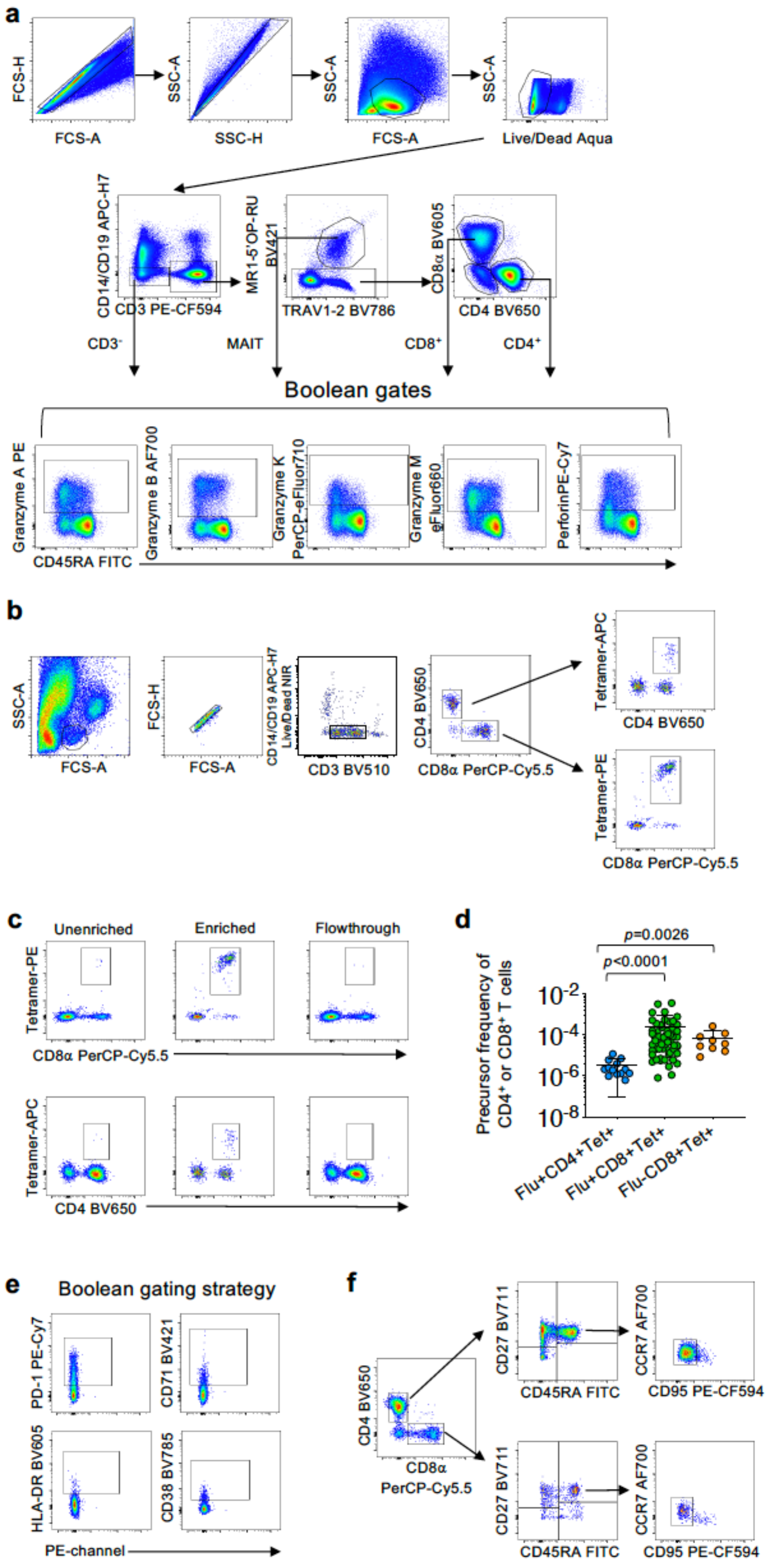
a
FCS-H
FCS-A
SSC-A
SSC-H
SSC-A
FCS-A
SSC-A
Live/Dead Aqua
CD14/CD19 APC-H7
CD3 PE-CF594
MR1-5'OP-RU BV421
TRAV1-2 BV786
CD8α BV605
CD4 BV650
CD3⁻
MAIT
CD8⁺
CD4⁺
Boolean gates
Granzyme A PE
Granzyme B AF700
Granzyme K PerCP-eFluor710
Granzyme M eFluor660
PerforinPE-Cy7
CD45RA FITC
b
SSC-A
FCS-A
FCS-H
FCS-A
CD14/CD19 APC-H7 Live/Dead NIR
CD3 BV510
CD4 BV650
CD8α PerCP-Cy5.5
Tetramer-APC
CD4 BV650
Tetramer-PE
CD8α PerCP-Cy5.5
c
Unenriched
Enriched
Flowthrough
Tetramer-PE
CD8α PerCP-Cy5.5
Tetramer-APC
CD4 BV650
d
p=0.0026
p<0.0001
Precursor frequency of CD4⁺ or CD8⁺ T cells
10-2
10-4
10-6
10-8
Flu+CD4+Tet+
Flu+CD8+Tet+
Flu-CD8+Tet+
e
Boolean gating strategy
PD-1 PE-Cy7
CD71 BV421
HLA-DR BV605
CD38 BV785
PE-channel
f
CD4 BV650
CD8α PerCP-Cy5.5
CD27 BV711
CD45RA FITC
CCR7 AF700
CD95 PE-CF594
CD27 BV711
CD45RA FITC
CCR7 AF700
CD95 PE-CF594

Supplementary Figure 6. TAME and phenotypic analysis of influenza-specific T cells. a, Boolean gating strategy for measuring each cell subset expressing a combination of granzymes A, B, K and M and perforin related to Fig. 5e-g. Data from PBMCs left over from flow through fraction following TAME. **b**, Representative FACS plots of enriched class I and class II tetramer populations following TAME related to Fig. 6a-f. **c**, Representative FACS plots showing the unenriched, enriched and flowthrough fractions of class I (top panels) and class II (bottom panels) tetramer^{+} populations related to Fig. 6a-f. **d**, Pooled tetramer^{+} precursor frequencies. Mean and SD are shown. Statistical significance ($0.0001>p<0.05$) was determined using Kruskal-Wallis test (two-sided). **e**, Boolean gating strategy of activation markers PD-1, CD71, HLA-DR and CD38 for CD4^{+}, CD8^{+} and tetramer^{+} T cell populations related to Fig. 6g-i. (F) Phenotypic gating strategy based on T cell differentiation markers related to Fig. 6j.

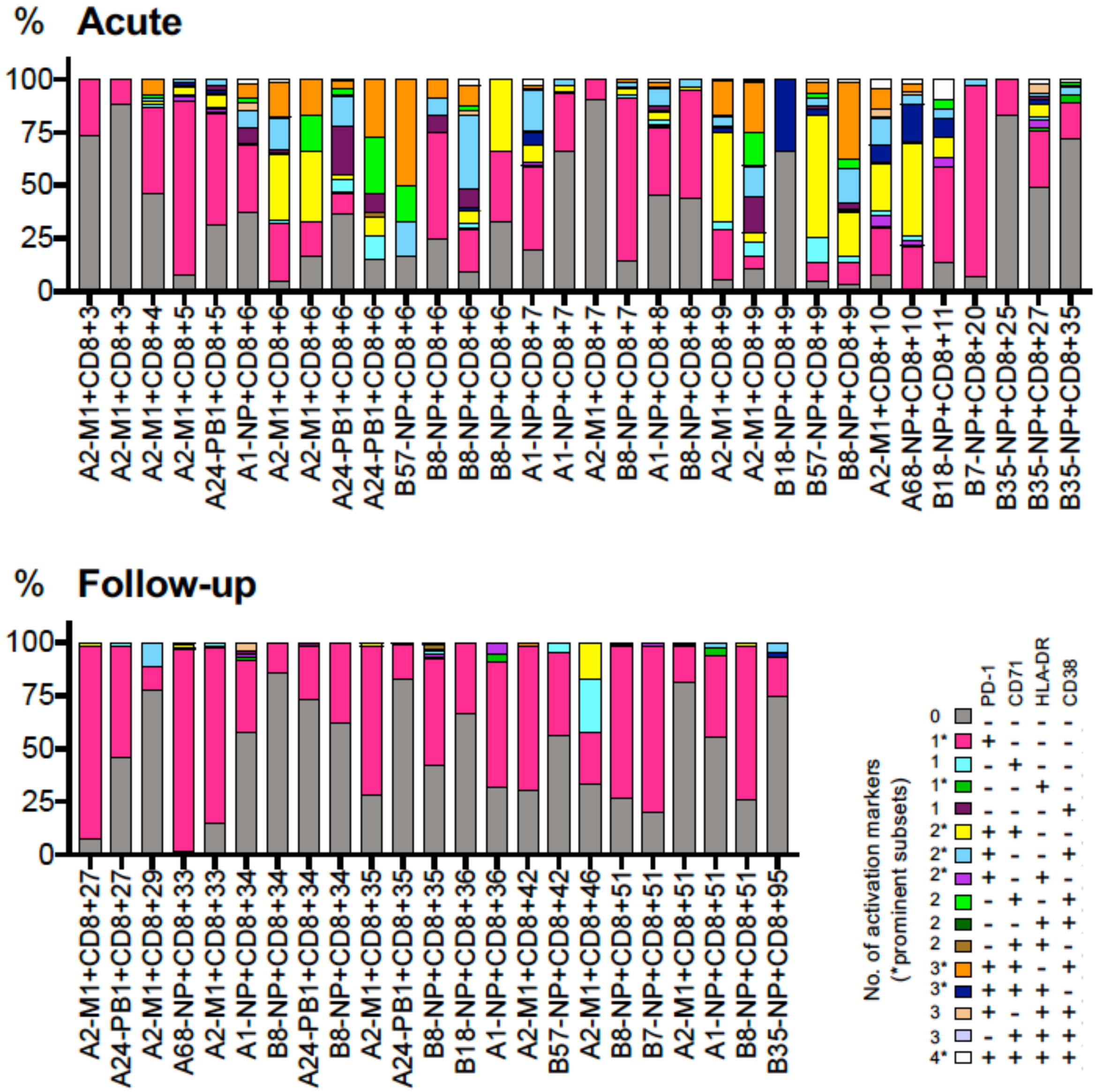

Supplementary Figure 7. Activation phenotype of class I-tetramer^{+} CD8^{+} T cells. Frequencies of activation markers on TAME-enriched tetramer^{+} cells at acute and follow-up timepoints. Patients are plotted individually and ordered along the x-axis by epitope+days of disease onset.

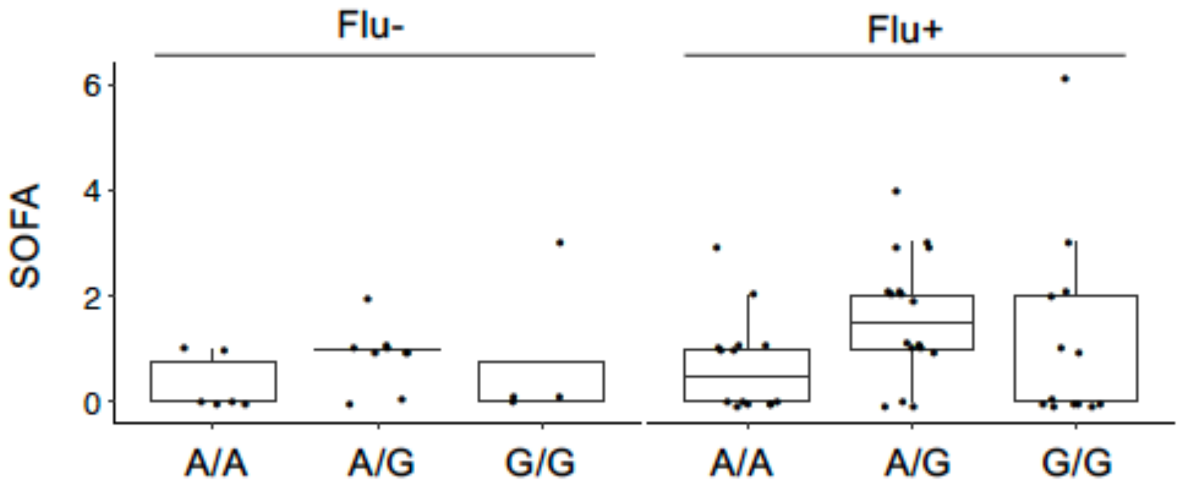

Supplementary Figure 8. No differences in IFITM SNPs and SOFA scores. Box plots of rs34481144 alleles against SOFA scores in influenza+ (*n*=44) and influenza- (*n*=20) patients showing median, IQR and whiskers extending to the largest or smallest values no further than 1.5 times the IQR.

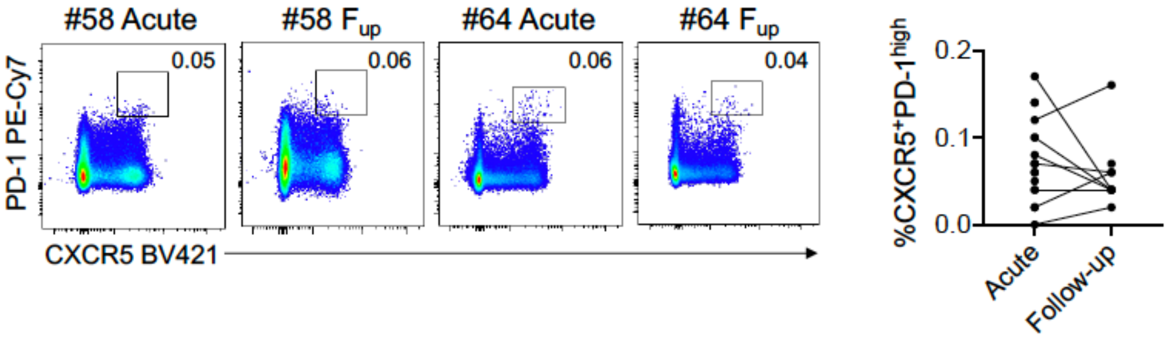

Supplementary Figure 9. CXCR5^{+}PD-1^{high} cTfh cells in the blood. Representative CXCR5 and PD-1^{high} expression and frequencies of CXCR5^{+}PD-1^{high} cTfh cells of total CD4^{+} T cells in influenza+ patients (*n*=14) at acute and follow-up (F_{up}) where available. Cells were gated on live/CD3^{+}/CD4^{+} T cells.

Supplementary Table 1. Patient summary.

	Influenza-positive, value	Influenza-negative, value
Sample size	44	20
Influenza A (%)	34 (77%)	
pH1N1, H3N2, unsubtyped	7, 24, 3	
Influenza B (%)	10 (23%)	
Phuket, Brisbane, unsubtyped	4, 3, 3	
RSV		3
Parainfluenza 1		3
Picornavirus		3
Other		11
Age, median (range)	54 (21-90)	47 (20-90)
Male (%)	25 (57%)	10 (50%)
Ethnicity		
White Australian	36 (82%)	19 (95%)
White Australian/Non Aboriginal/Torres Strait Islander	3 (7%)	
Non Aboriginal/Torres Strait Islander	3 (7%)	1 (5%)
Asian	1 (2%)	
Other (Sri Lankan)	1 (2%)	
Significant high-risk conditions*	38 (86%)	14 (70%)
Type of high-risk condition^		
Immunosuppressed	20	9
Chronic respiratory disease	27	8
Cardiac disease	6	1
Chronic renal disease	7	5
Liver disease	4	1
Neurologic disorders	6	1
Haematologic/malignancy disorders	2	1
Diabetes	8	5
Healthcare worker	2	1
Organ transplant recipient	2	
HIV-positive	3	2
Obesity	6	3
Vaccination status prior to infection		
Vaccinated in the year of infection	21 (48%)	13 (65%)
Vaccinated 1-2 seasons prior to infection	6 (14%)	3 (15%)
Vaccinated at least twice (year of infection and previous 1-2 seasons)	17 (39%)	11 (55%)
Total days in hospital, median (range)	4 (1-38)	4 (1-61)
Days from disease onset to admission, median (range)	4 (-4-23)	3 (1-14)
Days from disease onset to discharge/death, median (range)	8 (3-35)	10 (3-68)
Days from disease onset to follow-up, median (range)	41 (25-95)	39 (27-84)
Complications during acute illness		
Intensive care required	1	
Non-invasive support required	3	1
Pneumonia on clinical presentation	6	1
Bronchitis		1
Bronchiectasis	1	1
Secondary bacterial infection	1	
Bacterial growth in sputum or faecal	6	2
Exacerbation of COPD	1	1
Febrile neutropenia	1	1
Atrial fibrillation	1	
Tachypnoea		1
Death	1	1
Received antiviral therapy (%)	37 (84%)	9 (45%)
Received oseltamivir	36	9

*Persons with high-risk conditions defined by the Communicable Diseases Network Australia (CDNA) National Guidelines for Public Health Units: Seasonal Influenza Infection

^Any participant with one or more conditions

Supplementary Table 2. Panel of influenza viruses used in the antibody landscapes.

Group	Virus strain	Abbreviation	Vaccine component year (Australia)
H1N1	A/H1N1/Puerto Rico/8/34	PR/8/34	
	A/H1N1/Brazil/11/78	BRAZIL/11/78	
	A/H1N1/Fukishima/141/06	FUK/141/06	
	A/H1N1/Brisbane/59/07	BRIS/59/07	
	A/H1N1/California/07/2009	CAL/07/09	2014-16 vaccine
	A/H1N1/Michigan/45/2015	MICH/45/15	2017-18 vaccine
H3N2	A/H3N2/Port Chalmers/1/1973	PC/1/73	
	A/H3N2/Texas/1/1977	TEXAS/1/77	
	A/H3N2/Panama/2007/1999	PANAMA/2007/99	
	A/H3N2/Brisbane/10/2007	BRIS/10/07	
	A/H3N2/Perth/16/2009	PERTH/16/09	
	A/H3N2/Victoria/361/2011	VICTORIA/361/11	
	A/H3N2/Texas/50/2012	TEXAS/50/12	2014 vaccine
	A/H3N2/Victoria/2078/2014	VICTORIA/2078/14	
	A/H3N2/South Australia/91/2014	SOUTHAUS/91/14	
	A/H3N2/Victoria/3044/2014	VICTORIA/3044/14	
	A/H3N2/Newcastle/22/2014	NEWCASTLE/22/14	
	A/H3N2/Switzerland/9715293/2013	SWI/9715293/13	2015 vaccine
	A/H3N2/New Caledonia/71/2014	NEWCALED/71/14	
	A/H3N2/Brisbane/47/2015	BRISB/47/15	
	A/H3N2/Hong Kong/4801/2014	HONGKONG/4801/14	2016-17 vaccine
	A/H3N2/Singapore/INFIMH-16-0019/2016	SING/INFIMH-16-0019/16	2018 vaccine
B*	B/YAM/Brisbane/1/2007	BRIS/1/07	
	B/YAM/Massachusetts/02/2012	MASS/02/12	2014 TIV vaccine
	B/YAM/Phuket/3073/2013	PHUKET/3073/13	2015 TIV, 2016-2018 QIV vaccine
	B/YAM/Sydney/7/2014	SYDNEY/7/14	
	B/YAM/Brisbane/9/2014	BRIS/9/14	
	B/VIC/Brisbane/60/2008	BRIS/60/08	2016-2018 QIV vaccine
	B/VIC/Brisbane/46/2015	BRIS/46/15	

*TIV was substituted with the QIV in 2016 to incorporate both influenza B Yamagata and Victoria strains.

Supplementary Table 3. Healthy donors used in this study (Fig. 4, G to I).

Donor code	Age	Sex	Blood product	Source	MAIT cells measured
B124	49	M	Buffy pack	Australian Red Cross Lifeblood	Yes
B125	75	F	Buffy pack	Australian Red Cross Lifeblood	Yes
B126	27	M	Buffy pack	Australian Red Cross Lifeblood	Yes
B127	68	F	Buffy pack	Australian Red Cross Lifeblood	Yes
B142	20	F	Buffy pack	Australian Red Cross Lifeblood	N/A
B143	70	F	Buffy pack	Australian Red Cross Lifeblood	N/A
B144	36	M	Buffy pack	Australian Red Cross Lifeblood	N/A
B150	34	M	Buffy pack	Australian Red Cross Lifeblood	N/A
B153	29	M	Buffy pack	Australian Red Cross Lifeblood	N/A
B154	36	M	Buffy pack	Australian Red Cross Lifeblood	N/A
B155	35	F	Buffy pack	Australian Red Cross Lifeblood	N/A
B156	39	M	Buffy pack	Australian Red Cross Lifeblood	N/A
K26	29	M	Heparinized blood	Healthy volunteers	Yes
K28	35	F	Heparinized blood	Healthy volunteers	Yes
K99	21	F	Heparinized blood	Healthy volunteers	Yes
K100	22	F	Heparinized blood	Healthy volunteers	Yes
D8	78	M	Heparinized blood	Deepdene Surgery	Yes
D11	79	F	Heparinized blood	Deepdene Surgery	Yes
D13	87	F	Heparinized blood	Deepdene Surgery	Yes
D16	72	M	Heparinized blood	Deepdene Surgery	Yes

Abbreviations: F, female; M, male; N/A, not available.

Supplementary Table 4. HLA population coverage across ethnicities.

Average frequency (%)	Caucasoid*	Oriental*	African*	Amerindian*	Alaskan Yupik#	Australian Aboriginals*
HLA-A1	14.07	3.66	4.85	5.50	0.40	1.00
HLA-A2	25.01	27.17	15.76	24.78	2.40	7.85
HLA-A24	10.36	23.97	3.14	30.90	58.10	48.75
HLA-A68	3.99	1.29	9.68	5.95	10.10	1.50
HLA-B7	8.67	3.37	7.71	2.38	1.60	0.75
HLA-B8	7.41	1.40	4.83	1.10	0.40	0.50
HLA-B18	6.31	0.92	4.62	0.50	0.60	0.00
HLA-B35	10.33	5.03	5.53	17.53	10.70	1.75
HLA-B57	2.91	1.33	3.96	0.68	0.00	1.75
HLA-DR1	9.42	2.98	5.46	1.5	0.40	1.00^
HLA-DR4	12.82	12.99	10.51	40.00	23.20	0.50^
HLA-DR11	13.36	7.74	15.74	11.65	10.70	N/A
Total	>100	91.85	91.79	>100	>100	63.85

Percentages based on HLA coverage for the relevant HLA supertype. N/A, not available.
*Marsh SGE, et al. (2000) The HLA Factsbook (Academic Press, San Diego).
#www.allelefrequencies.net.
^Australia Cape York Peninsula Aborigine (www.allelefrequencies.net).

Supplementary Table 5. List of primers.

Primer	Forward (F) 5'-3'	Reverse (R) 5'-3'
IFITM3 amplification*	GGAAACTGTTGAGAAACCGAA	CATACGCACCTTCACGGAGT
rs34481144 sequencing	ACAGCCACCTCGTGCTCCTC	GTTGAGAAACCGAAACTACTGGG
A-HA-Uni	GGGGGGAGCAAAAGCAGGGGA	CCGGGTTATTAGTAGAAACAAGGG TG
B-HANA-Uni3	GGGGGAGCAGAAGCAGAGC	CCGGGATATTAGTAGTAACAAGAG C

*Primers were used to amplify both *exon 1* rs12552 and rs34481144 promoter regions. The same primers were used to sequence rs12252.
