# Supplementary material for: Immune cellular networks underlying recovery from influenza virus infection in acute hospitalized patients
Source: Nat Commun. 2021 May 11;12:2691. doi: 10.1038/s41467-021-23018-x (PMC8113517; doi:10.1038/s41467-021-23018-x)
Supplement: Supplementary file 3 — Description of Additional Supplementary Files [file 41467_2021_23018_MOESM3_ESM.pdf]

Description of Additional Supplementary Files

File Name: Supplementary Data 1
Description: Individual patient demographics and HLA typing.xls

File Name: Supplementary Data 2
Description: Interactive unsupervised heatmaps of acute influenza+.html

File Name: Supplementary Data 3
Description: Interactive unsupervised heatmaps of convalescent influenza+.html

File Name: Supplementary Data 4
Description: Interactive unsupervised heatmaps of acute influenza-.html

File Name: Supplementary Data 5
Description: Interactive unsupervised heatmaps of convalescent influenza-.html

File Name: Supplementary Data 6
Description: Interactive unsupervised heatmaps of full influenza+ and influenza- cohort.html

File Name: Supplementary Data 7
Description: Patient sample used for each assay.xls

File Name: Supplementary Data 8
Description: Antibody panels used for flow cytometry.xls
