# Supplementary material for: Immune cellular networks underlying recovery from influenza virus infection in acute hospitalized patients
Source: Nat Commun. 2021 May 11;12:2691. doi: 10.1038/s41467-021-23018-x (PMC8113517; doi:10.1038/s41467-021-23018-x)
Supplement: Supplementary file 12 — Reporting Summary [file 41467_2021_23018_MOESM12_ESM.pdf]

nature research

Corresponding author(s): Katherine Kedzierska

Last updated by author(s): Jan 28, 2021

Reporting Summary

Nature Research wishes to improve the reproducibility of the work that we publish. This form provides structure for consistency and transparency in reporting. For further information on Nature Research policies, see Authors & Referees and the Editorial Policy Checklist .

Statistics

For all statistical analyses, confirm that the following items are present in the figure legend, table legend, main text, or Methods section.

n/a	Confirmed	
☐	☒	The exact sample size (*n*) for each experimental group/condition, given as a discrete number and unit of measurement
☐	☒	A statement on whether measurements were taken from distinct samples or whether the same sample was measured repeatedly
☐	☒	The statistical test(s) used AND whether they are one- or two-sided<br>*Only common tests should be described solely by name; describe more complex techniques in the Methods section.*
☐	☒	A description of all covariates tested
☐	☒	A description of any assumptions or corrections, such as tests of normality and adjustment for multiple comparisons
☐	☒	A full description of the statistical parameters including central tendency (e.g. means) or other basic estimates (e.g. regression coefficient) AND variation (e.g. standard deviation) or associated estimates of uncertainty (e.g. confidence intervals)
☐	☒	For null hypothesis testing, the test statistic (e.g. *F*, *t*, *r*) with confidence intervals, effect sizes, degrees of freedom and *P* value noted<br>*Give P values as exact values whenever suitable.*
☒	☐	For Bayesian analysis, information on the choice of priors and Markov chain Monte Carlo settings
☒	☐	For hierarchical and complex designs, identification of the appropriate level for tests and full reporting of outcomes
☒	☐	Estimates of effect sizes (e.g. Cohen's *d*, Pearson's *r*), indicating how they were calculated

Our web collection on statistics for biologists contains articles on many of the points above.

Software and code

Policy information about availability of computer code

Data collection	BD FACS DIVA v8.0.1
Data analysis	FlowJo v9, 10; Prism v8.3; R v3.5.3; Pestle v1.8, Spice v5, Geneious 9.0.4

For manuscripts utilizing custom algorithms or software that are central to the research but not yet described in published literature, software must be made available to editors/reviewers. We strongly encourage code deposition in a community repository (e.g. GitHub). See the Nature Research guidelines for submitting code & software for further information.

Data

Policy information about availability of data

All manuscripts must include a data availability statement. This statement should provide the following information, where applicable:

- Accession codes, unique identifiers, or web links for publicly available datasets
- A list of figures that have associated raw data
- A description of any restrictions on data availability

All data generated or analysed during this study are included in this published article (and its supplementary information files). Source data are provided with this paper. All relevant data are also available from the authors. The viral sequences isolated from nasal swabs that support the findings of this study are available on the GISAID database with ID numbers of EPI_ISL_312219-312221, 312673-312674. Registration to access the database is free and open to anyone using the link: https://www.gisaid.org/registration/register/.

Field-specific reporting

Please select the one below that is the best fit for your research. If you are not sure, read the appropriate sections before making your selection.

☒ Life sciences ☐ Behavioural & social sciences ☐ Ecological, evolutionary & environmental sciences

For a reference copy of the document with all sections, see nature.com/documents/nr-reporting-summary-flat.pdf

Life sciences study design

All studies must disclose on these points even when the disclosure is negative.

Sample size	The sample size was determined by the availability of samples collected during 2014-2017. This is a longitudinal influenza patient cohort.
Data exclusions	No data were excluded with the following exception which was pre-established: donors who had a total number of less then 10 counted tetramer+CD8+ or tetrame+CD4+ T cells within the whole enriched fraction (Fig. 6) were excluded for further phenotypic analyses as cell numbers were too low. This was indicated in the manuscript under methods.
Replication	Experiments could not be replicated due to limited PBMC numbers. These are rare and precious patient samples and so we were limited to performing all the available assays. To ensure reliability, all timepoints from the same patient were carried out in the same experiment.
Randomization	Randomization was not applicable to the study. The study recruited case patients followed by the next available control patient. Where no controls were available, then cases were still recruited. Other covariates were not controlled in the study as we did not exclude anyone willing to participate in the study.
Blinding	Experiments were not blinded as specific experiments were designed for individual case and control patients, for example the flu status and cell availability was needed to perform certain assays, their flu status and HLA type was needed for the tetramer studies, the Influenza infection study was an overnight culture assay and so HIV status was also important to know.

Reporting for specific materials, systems and methods

We require information from authors about some types of materials, experimental systems and methods used in many studies. Here, indicate whether each material, system or method listed is relevant to your study. If you are not sure if a list item applies to your research, read the appropriate section before selecting a response.

Materials & experimental systems

n/a	Involved in the study	
☐	☒	Antibodies
☒	☐	Eukaryotic cell lines
☒	☐	Palaeontology
☒	☐	Animals and other organisms
☐	☒	Human research participants
☒	☐	Clinical data

Methods

n/a	Involved in the study	
☒	☐	ChIP-seq
☐	☒	Flow cytometry
☒	☐	MRI-based neuroimaging

Antibodies

Antibodies used	We used commercially-available antibodies as per Material and Methods, and Supplementary Data 8.
Validation	Each antibody used had a validated technical data sheet as per manufacturer's website showing positive staining. The catalogue numbers and manufacturer's details are listed in Supplementary Data 8. All antibodies were titrated in our laboratory prior to their use and positive staining is shown for each antibody used as shown in the FACS plots in the main figures and supplementary figures.

Human research participants

Policy information about studies involving human research participants

Population characteristics	Please refer to Supplementary Data 1 (individual patient characteristics), Supplementary Tables 1 (summarized patient characteristics) and 3 (healthy donors) for further details.
Recruitment	Healthy control samples were recruited through The University of Melbourne (blood donors), Australian Red Cross Lifeblood (buffy packs) and Deepdene Surgery (healthy elderly adult blood donors through co-author J. Crowe). Patients admitted to The Alfred Hospital with influenza-like-illness were recruited to the study through co-authors A Cheng and T Kotsimbos. Signed informed consents were obtained from all blood donors prior to the study. There was no self-selection bias or any other bias for recruiting donors to the study.
Ethics oversight	Human experimental work was conducted according to the Declaration of Helsinki Principles and according to the Australian National Health and Medical Research Council Code of Practice. Participants provided written informed consent prior to the study. The study was approved by the Alfred Hospital (ID #280/14) and University of Melbourne (ID #1442952.1 and #1443389.4) Human Research Ethics Committees.

Note that full information on the approval of the study protocol must also be provided in the manuscript.

Flow Cytometry

Plots

Confirm that:

- [x] The axis labels state the marker and fluorochrome used (e.g. CD4-FITC).
- [x] The axis scales are clearly visible. Include numbers along axes only for bottom left plot of group (a 'group' is an analysis of identical markers).
- [x] All plots are contour plots with outliers or pseudocolor plots.
- [x] A numerical value for number of cells or percentage (with statistics) is provided.

Methodology

Sample preparation	Samples were prepared as described in Methods. For Figure 3, whole blood was used for direct staining of immune cell subsets for flow cytometry. PBMCs were isolated from the sodium heparinized blood or buffy packs using Ficoll-Paque (GE Healthcare, Uppsala, Sweden) density-gradient centrifugation. The mononuclear layer was harvested and cells were washed by centrifugation before being cryopreserved. PBMCs were thawed and immediately used in the assays for Figures 3-6.
Instrument	BD LSRII Fortessa and BD Canto II were used for acquisition of data.
Software	BD FACS Diva v8.0.1, FlowJo v9 and v10.5.3
Cell population abundance	We have not sorted the PBMCs.
Gating strategy	Gating strategy has been described in the figures, figure legends and supplementary figures 2, 5 and 6.

- [x] Tick this box to confirm that a figure exemplifying the gating strategy is provided in the Supplementary Information.
